# Smartphone-Based Biosensor Devices for Healthcare: Technologies, Trends, and Adoption by End-Users

**DOI:** 10.3390/bioengineering9030101

**Published:** 2022-03-01

**Authors:** Rossana E. Madrid, Fernando Ashur Ramallo, Daniela E. Barraza, Roberto E. Chaile

**Affiliations:** Laboratorio de Medios e Interfases (LAMEIN), DBI, FACET, Universidad Nacional de Tucumán, Instituto Superior de Investigaciones Biológicas (INSIBIO), CONICET, Av. Independencia 1800, San Miguel de Tucuman 4000, Argentina; ferchoashur@gmail.com (F.A.R.); barrazadaniela86@gmail.com (D.E.B.); rechaile@gmail.com (R.E.C.)

**Keywords:** POC devices, electrochemical biosensors, paper-based biosensors, optical biosensors, commercial biosensors, regulations

## Abstract

Smart biosensors are becoming an important support for modern healthcare, even more so in the current context. Numerous smartphone-based biosensor developments were published in recent years, some highly effective and sensitive. However, when patents and patent applications related to smart biosensors for healthcare applications are analyzed, it is surprising to note that, after significant growth in the first half of the decade, the number of applications filed has decreased considerably in recent years. There can be many causes of this effect. In this review, we present the state of the art of different types of smartphone-based biosensors, considering their stages of development. In the second part, a critical analysis of the possible reasons why many technologies do not reach the market is presented. Both technical and end-user adoption limitations were addressed. It was observed that smart biosensors on the commercial stage are still scarce despite the great evolution that these technologies have experienced, which shows the need to strengthen the stages of transfer, application, and adoption of technologies by end-users.

## 1. Introduction

When one thinks of POC devices, the literature always refers to the development of low-cost health care technology with low-income populations in mind or places with difficult access. However, in recent years, and more so in the present pandemic context, it can be seen that having POC devices everywhere would greatly help the management and care of patients and health personnel.

**Figure 1:**
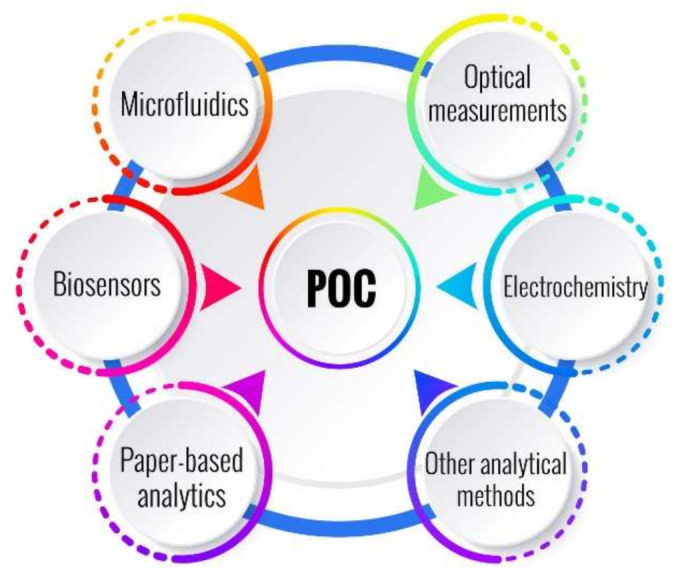
Generalization of the Venn diagram proposed by Rackus et al. [1]. It shows the interaction of biosensors, microfluidics, and different technologies and analytical methods, which gives rise to POC devices.

Different disciplines can converge into POC devices development, such as chemistry, biology, physics, and engineering; their combination gives rise to an interesting variety of sub-disciplines, such as biosensors, biochips, and microfluidics. When microfluidics is combined with biosensors, the possibilities become limitless. The integration of both technologies provides the possibility of miniaturized devices, an important and highly sought feature for the development of POC devices. Rackus and collaborators propose a Venn diagram, an interesting scheme that summarizes this concept [1]. They show how these sub-disciplines overlap and work together, and they argue, quite appropriately, that the overlapping of these three fields gives rise to point-of-care systems. However, if electrochemistry is changed by optical or other types of biosensors, this combination is an interesting example of how they are combining to form new application areas, which reveals great opportunities to develop POC devices. Figure 1 show this idea by modifying the first Venn diagram proposed by Rackus et al. It also includes the use of new materials, such as paper-based chips, new fabrication procedures, and different analytical methods.

On the other hand, in the last decade, there was an explosion of smartphone-based biosensors [2,3,4]. The ubiquity of smartphones throughout the world has brought about new opportunities to bring POC devices near the patients for portable healthcare monitoring, taking advantage of the characteristics of computing power, network connectivity, battery, and cameras of these devices. This can help both patients and physicians for the faster, more efficient and reliable resolution of any health problem that may arise at home or outside the context of healthcare centers. In addition, the widespread connectivity options of the current wireless telecommunication infrastructure make the smartphone a ubiquitous platform worthy of using in order to develop biosensing and diagnostics platforms, especially for point-of-care and telemedicine applications. POC devices help to bring diagnoses closer to the patient by providing faster and more frequent feedback with the physicians [5]. The latter is the *raison d’être* of smart POC biosensors.

Wearable biosensors are also important to consider but deserve special consideration, so they will only be considered if there are any special cases. Readers who are interested in this particular topic can refer to very complete and excellent reviews in the bibliography such as those of Ray et al. [6]; Kim et al. (2015 and 2018) [7,8]; Ajami and Teimouri [9]; Bandodkar et al. [10]; Nag et al. [11]; Tamsin [12]; Rodrigues et al. [13]; Chung et al. [14]; Lee et al., to name only a few.

Electrochemical biosensors are undoubtedly the most popular among POC devices due to their high sensitivity, simplicity, low cost, and reliability. The development of these electrochemical devices has continued to grow exponentially since Clark designed the first enzymatic glucose biosensor [15], which was then improved and became the most commercialized healthcare biosensor. In the last five years, electrochemical biosensors that use smartphones received great attention as they use a friendly semi-automated user interface with minimum extra tailored hardware. They can also be used at home, offering an interesting, cost-effective alternative. A very interesting review by Sun and Hall presents a study on the different technologies used in electrochemical smartphone-based biosensors in terms of the voltage sources used, the power required in each case, and the resolution and detection limit characteristics [5].

Optical biosensors also showed significant growth in recent years, even more so with the use of smartphones that allow their use as transmitters or receivers of optical signals. On the other hand, the introduction of paper as a substrate for the development of analytical systems proved to be the most chosen in recent years. This type of substrate allows for the implementation of both electrochemical and optical biosensors.

Microfluidic paper-based analytical devices (µPAD) applied to biosensing technologies were widely developed since their first proposal by the Whitesides group in 2007 [16]. Paper possess networks of hydrophilic/hydrophobic micro channels, which make quantitative analysis possible for their potential application in biochemical environments in healthcare. Furthermore, focusing on the point-of-care approach, paper-based sensing devices were connected with optical or colorimetric reactions in order to obtain rapid and on-site results. However, paper also presents some limitations, such as reproducibility and repeatability, and the measurements are more difficult to automate. These limitations impact the quality of the results, mainly regarding naked-eye detections where the operator may have subjective interpretation on different results. In order to tackle these limitations for paper-based optical devices and improve their outcome, in recent years, these devices were combined with smartphone technologies to capture, analyze, and quantify analytical measurements, having a better and more robust performance [17].

However, do all biosensor developments actually reach the patients? This review will make an evaluation of the smartphone-based biosensors that did reach a commercial instance by also evaluating those which have a patent or patent application to present the current state of the art trends in these sensor technologies. The latest reports considered in the last five years were included, and the review was divided into two main sections. The first one presents the state of the art for different types of smartphone-based biosensors considering their stages of development. In all cases, examples were considered where the developed device was closest to a commercial prototype and particularly in those that were evaluated with real samples. The second section presents a critical analysis of the possible reasons why many technologies do not reach the market and the steps the technology should take to reach patients.

The bibliography was explored looking for smart devices that use smartphones as smart interfaces, either to obtain images that will later be processed, which are modified to read a device on the same phone, or where the phone is used to transmit the data to service centers. Almost 550 articles were reviewed in the Scopus search engine for the search result “biosensor + smartphone + POC”, in general, and then for particular applications with the search “electrochemical + smartphone + biosensor” or “biosensor + smartphone + paper-based”, or “optical + smart + biosensor”. The papers were reviewed from January 1, 2015, to December 2021; however, some articles outside these dates were also included when deemed appropriate. In total, 22.5% of the reviewed papers were included.

Patent databases were explored for technologies, inventors, and institutions to correlate publications with patented technologies. Finally, the search for commercial biosensors and for the regulations that devices must comply with to move to the commercial stage was facilitated in an internet exploration.

## 2. Current State of Art and Trends in Smartphone-Based Sensors Field

**Figure 2:**
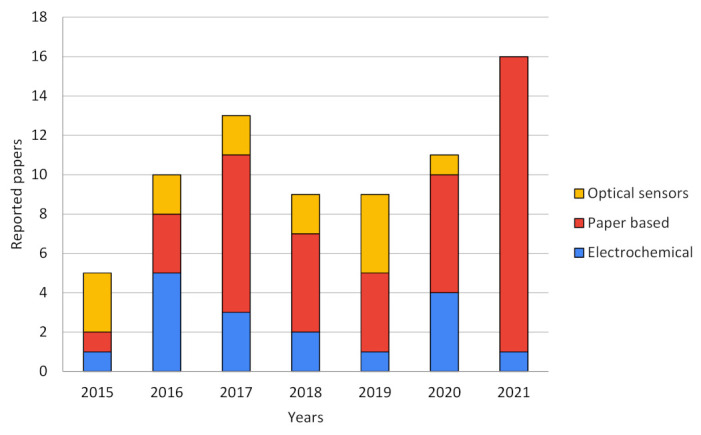
Publications trend of smartphone-based biosensors over the years by type of technology.

Both scientific research articles and patent databases were consulted in order to elucidate the current state of the art trends in smartphone-based sensors technologies. In order to simplify the classification of each reviewed paper or patent, technologies were segmented into three categories: (A) electrochemical sensors, (B) optical sensors, and (C) paper-based sensors. Figure 2 show the trends in each technology over the years reviewed, expressed in numbers of published papers related to smartphone-based sensors.

As can be seen in Figure 2, in the last five years, the popularity of smartphone-based sensors, measured as the number of publications, increased in general and is doubtless linked to the rapid evolution and development of smartphones due to their processing power and the better performance of their tools such as cameras and light sensors [18,19]. It can be seen that electrochemical and optical sensors were featured in most of the publications until approximately 2016, but further and near 2020 and 2021, paper-based sensors mainly occupy the major scene in this field. This evolution trend can be explained due to the type of strategy used by researchers when profiting the smartphone features. Electrochemical sensors use smartphones not only as point-of-care potentiometric devices and signal processing but also as the power source of the whole biosensor. Optical devices generally need specific appliances, hardware, and a complex isolated environment in order to achieve good results. Some of these drawbacks favor paper-based electrochemical proposals due to their cheaper fabrication and simpler setups to achieve comparable results to pure electrochemical devices. As for pure optical sensors, a fairly stable development can be observed over the years reviewed. This could be due to the complexity of the optical systems required, which have apparently been replaced by paper-based optical sensors that take advantage of the advent of better cameras, improved light sensors, and more powerful image processing systems in smartphones [20].

**Figure 3:**
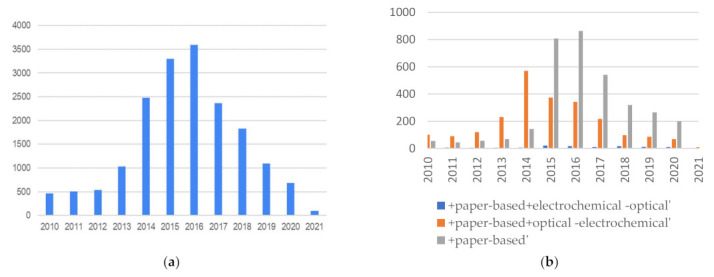
(**a**) Patent applications “biosensor + smartphone + poc(point-of-care)”; (**b**) Patent applications for electrochemical, paper-based, and optical smartphone based biosensors.

When analyzing overall patent applications by filtering in a wider time window, a very interesting response can be seen regarding patent applications with the search pattern “biosensor + smartphone + point-of-care” or “biosensor + smartphone + poc”. As can be seen in Figure 3, there is a systematic increase in patents from 2010 to around 2016 and then a sharp decrease until 2021. It is interesting to see how smart devices became popular until 2016, but the significant decrease in recent years is striking.

Figure 3b show the same data but discriminates between the different types of biosensors. The same trend can be observed. The large increase in patent applications in this area in the first half of the decade until 2016 correlates with the advancement of smartphone technology, but it is surprising why, in the second half, they declined rapidly. Perhaps the “Theranos effect” may have played a role that was not minor. On the other hand, frequently, patent applications are carried out with laboratory validations, but later, moving on to the technology implementation stage and application with real samples becomes more difficult. Another fact to consider is that some publications on biosensors are developed on devices that are already widely used, such as those of glucose, for example, and the novelty to make them smart is not enough to achieve a technology replacement by the users. It is expected that the publications of the last years will be delayed since the development of new devices and innovation in the area has grown a lot and, as is known, patents are filed first. Therefore, in this case, the patent applications decreased in recent years.

Another interesting fact is that this effect is not evident for electrochemical sensors. Despite their popularity, they are much less prevalent, but they remain in number throughout the reviewed period.

## 3. Overview of Reviewed Technologies by Type of Sensors and Commercial Stage

This section presents an overview of the different types of smartphone-based biosensors, taking into account the transduction method and the substrate material. In this way, biosensors were classified as electrochemical, optical, and paper-based biosensors.

### 3.1. Electrochemical Smartphone-Based Biosensors

The integration of electrochemical POC devices with smartphones is a very promising strategy due to the great improvement of the advantages of each technology. Electrochemical biosensors have high sensitivity and specificity, with the possibility of simple and fast quantitative measurements, all features that can be enhanced with the use of smartphones.

Numerous strategies are used in the development of this type of device, using, for example, smartphones as the electrochemical analyzer or simply to power external dongles. This is an important feature to take into account, that is, the way the measurement module is integrated into the smartphone [5]. There are wired peripherals, for example, through the USB with OTG (On-The-Go) protocol (a kind of device communication standard), which limits its use depending on the model and brand of the phone or the ones that use the audio headphone port. The wireless peripherals (where the connection is via Bluetooth and near-field communication (NFC)) have the benefits that the measurement electrodes can be integrated near the patient, even being wearable, and the smartphones can be a potential source of energy, signal processing, and are convenient devices for data readout in wearables [21]. The case of internal dedicated hardware is another method of integration with the smartphone, and although there are some examples of them [22,23], the main problem is that developments of this type are made for a particular type of smartphone and therefore are restricted only to that particular type and brand of phone [5].

Considering the most recent reports on this topic, some examples of electrochemical biosensors that use smartphones are here presented. Table 1 presents the selected publications of the last six years considering the publications that have patents or patent applications, which gives an indication of which technologies would go to the next stage of the application and use in patients. As can be seen, only one-third of the selected publications have application or granted patents.

The SARS-CoV-2 outbreak, which rapidly evolved into a worldwide pandemic, is an example of a very important event, where smart POC biosensors have become of vital importance to manage the disease and avoid oversaturate health services. Some authors presented interesting mini-reviews of the development of POC biosensors for the detection of COVID-19, where numerous biosensors reported in the bibliography were analyzed and proposed to be perfectly applied in the detection of this new disease with the adequate adaptation of bioreceptors [38,39,40,41,42]. In this sense, electrochemical and optical biosensors would be the best suited to implement COVID-19 POC detection [38]. POC biosensors can provide valuable data for the effective assessment of clinical progress of the symptoms and to provide alertness on the severity or critical trends of infection. Moreover, if these devices are associated with smartphones or direct communication systems with health centers, unnecessary transfers could be avoided, and it would be possible to act more quickly on patients with a poor evolution. Table 1 reflect two examples of smartphone-based electrochemical biosensors for this application. Reliable biosensors that patients can buy in a pharmacy and make the determination at home will be very useful. Moreover, it seems convenient to develop biosensors to determine other useful parameters that, together with pulse oximetry determinations, avoid the unnecessary transfer of patients to hospitals or health care centers. Examples of these are the biosensor proposed by Miripour et al. for the detection of ROS species [35] or that of Baraket et al., who already in 2017, proposed a biosensor for the detection of cytokines [43]. Non-cytokine protein biomarkers such as C-reactive protein and D-dimer (a small protein fragment present in the blood after a blood clot is degraded by fibrinolysis, which is elevated in patients with COVID-19) or other biomarkers that can also be found in whole blood, serum, urine, saliva, or sweat, can also be used as important biomarkers for monitoring the disease at home. The connection of these biosensors to smartphone systems would allow not only remote control by doctors but also the protection of all health personnel and the general population.

Taking into account all applications of electrochemical smartphone-based pure electrochemical biosensors, it can be seen from Table 1 that only one-third of the reviewed papers were found to have patents or related ones. This may be due to many factors, from little practice of patenting in the countries where the works come from to difficulty in the effective transfer of technology to the market, or the lack of clinical importance of the detected analytes from a POC detection point of view.

### 3.2. Optical Smartphone-Based Biosensors

The following examples illustrate some of the most remarkable proposals regarding this area, presented in the literature between 2015–2020. The use of microscopy in order to achieve optical detection of biosensing and diagnostic devices is the most common strategy since it provides reliable information and on-site results compatible with point-of-care devices. Nevertheless, microscopy devices are high-quality performance equipment that present some inescapable requirements such as proper infrastructure for its size, high qualified operators, and sometimes high-cost supplies. On the other hand, image analysis for the transduction and quantification of radiation emission or color amount cast by analyte recognition demands using dedicated software in order to obtain information from a sample. For several years, most of these informatics tools were only driven on personal computers or specific equipment, but with the explosive development of mobile applications and rapid enhancement of the mobile processors and computing capacity, the analyzing tools are nowadays within easy reach.

In order to keep using the benefits of microscopy techniques, using image analysis tools, and looking forward to the point-of-care approach, these authors used convenient smartphone features to sense and diagnose biological analytes. Table 2 illustrate some of the most remarkable proposals regarding this area, presented in the literature in the mentioned period.

The great variety of optical biosensors reported in the bibliography saw their possibilities grow with the incorporation of smartphones as reading devices, actuators, image processors, or connections with the cloud. This incorporation made them very promising devices. In the present case, almost 100% of the publications are supported by patents, so most of them are nearer to a commercial prototype. On the other hand, only a few examples of smartphone-based optical biosensors were presented in Table 2 following the mentioned criteria, since most of the reports correspond to paper-based POC devices, which have even more possibilities and will be treated in the following section of this work.

### 3.3. Paper-Based Biosensors That Uses Smartphones

In recent years, paper has become an alternative for advanced microfluidic devices, being used as a platform for various analytical and bioanalytical techniques. Within the large volume of POC devices for health care that exists in the market, paper-based biosensors are the most chosen by end-users. Qualities such as their price and their robustness have allowed paper-based POC biosensors to distinguish themselves from other biosensors systems. A market analysis performed by “Grand View Research, USA”, evidenced that the participation of said diagnostic devices in 2016 was approximately $2.2 billion, and it was predicted that its participation would reach $8.35 thousand million for the year 2022 [67]. Together with qualities such as portability, functionalization and modification, lower cost, ease of manufacturing and transportation, profitability, and biodegradability, these devices recently achieved the SAFE status (affordable, sensitive, specific, easy to use, fast and robust, without equipment, deliverable to all end-users) for POC diagnostics in miniaturized environments [67,68] .

Depending on the complexities of fluid handling and precision, paper-based biosensors are classified into dipstick, side-flow assay (LFA), and µPAD, the last one being the only one capable of making a quantitative diagnosis. Due to all the aforementioned benefits of paper-based devices and to allow them to make quantitative or semi-quantitative estimates [69], research was promoted in recent years on their use as POC assisted by smartphones, strips readers, dedicated electronic devices, signal processing modules, etc. In this way, the development of high-quality peripheral-assisted diagnostic devices and the possibility of generating, at a lower cost, authenticated and organized records for future reference are also promoted [67,70].

In this sense, the use of smartphones is a leader over other smart devices for paper-based biosensors due to their easy handling, adjustment, and simplicity for end-users. The joint work of major smartphone manufacturers and healthcare giants has resulted in an overwhelming emergence of smartphone-based diagnostic devices for general health and fitness in this area [67]. In this way, among the paper-based devices that use intelligent technology, there are those that perform determinations by electrochemiluminescence, electrochemistry, and the most popular, optical measurements.

In the search carried out since 2015, almost 40 publications were found that met the search criteria “+smartphone + point-of-care + paper-based + biosensors”. However, despite the great advantages that these devices present, of all the reports reviewed, only one-third of them had patent applications or granted or related patents. The Table 3 resumes the most recent published papers that deals with paper-based smart devices, according to the type of analyte to be determined (biochemical analysis, immunoassays, and molecular diagnostics to detect DNA and other biomolecules), as was classified in the paper of Xu et al. [71], and according to the detection method (optical, electrochemical and electrochemiluminescent). The type of biological sample where the measurements are made is also highlighted. The selection of the papers to include in the Table 3, was made considering only those that have patents, as it was considered that they would be closest to a real field application device.

It is expected that the number of reports on the development of paper-based smart biosensors will increase and will take a stellar role not only in this pandemic but also in many applications for healthcare. However, although the amount of this type of device on the market and within reach of the people is beginning to increase, it is still scarce.

## 4. Sensors at Commercial Stage

As previously shown, in spite of all the benefits each type of smartphone-based biosensors presents, there is a downwards trend both in the particular and the overall analysis of patent applications throughout the 2015–2021 period. There are a few possible explanations that, together, might help shed light on this situation. First, the question of whether the expectations the devices generate can be met. This becomes especially important in the transfer process, and it is important that researchers maintain a realistic and sincere standpoint in front of possible investors. A formidable counterexample is the case of Theranos, a company that promised a device capable of performing a plethora of tests with a drop of blood. The promise was an exaggeration of the technology’s real capabilities, but the idea of portability in some diagnostics is oftentimes easier thought than implemented as many problems not present in a laboratory environment can simply trump the utility of the device when taken to a real-life environment. A variety of these can be considered, from the inability to isolate the signal from noise when it involves on-patient measuring to low adoption due to a steep learning curve to the use of some biosensors.

During this investigation, it was found with a considerable frequency, businesses offer, erroneously, products capable of detecting and measuring biological signals as biosensors, disregarding the definition of a biosensor, i.e., a device comprising a biological recognition element coupled with a physical transducer. Such is the case of Philip’s “Wearable biosensor” [87], which had great media coverage in light of being used as a complementary monitoring device for COVID-19 patients.

This section presents relevant commercially available POC devices marketed as smart and biosensor based, though some of them may not fall in line with the strict definition of a biosensor. The inclusion of relevant non-biosensor devices will provide a broader image of the market in which biosensor-based smart POC devices must be inserted.

Among biosensors, glucose biosensors may be the most studied and developed ones, with many devices being commercialized for decades. More recently, systems such as Senseonic’s Eversense CGM [88] have sought to reduce the patient’s involvement in the measurements, avoiding recurrent pricklings in the way. The system comprises an implantable glucose sensor that can last up to 90 days under the patient’s skin, sending information to an adhesive-like reader just above the skin. The sensor not only processes data and sends it to the patient’s phone but is also able to give on-body vibe alerts following smart tendency profiles, to warn the user of upcoming dangerous glucose levels. The companion mobile app shows real-time glucose measurements and allows the patient to record additional information such as exercise or meals, as well as share data with up to five people. The patent of the system was just granted in September 2021 [89].

On the other hand, there is an increasing interest in obtaining measurements in less invasive and painless ways. In this direction, Nemaura Medical’s sugarBEAT [90], which is yet to start selling, is a continuous glucose monitoring device comprising a discardable adhesive patch of daily use and a rechargeable transmitter that communicates with the user’s phone via Bluetooth. Measurements are made with interstitial fluid from the first layer of skin, and once processed by a proprietary algorithm, they are correlated with glucose levels. An app on the user’s phone shows data every 5 min and allows to manually enter diet, medication, exercise, and other related info to help understand how they all impact glucose levels. The app will also act as a relay to a support platform with personalized insights and recommendations called BEATdiabetes, for better management of the disease. Furthermore, Nemaura Medical claims that BEAT is a versatile platform that can be fitted to many other metabolites, such as lactate or alcohol.

Ingestible biosensors recently received a great deal of attention with a relative development maturity. The etectRx’s iD Capsule, for example, has completed the clinical trials in healthy volunteers, but the clinical trial is still ongoing [91]. The company has an active patent [92]. On the other hand, Proteus’ “smart pill” project, which seemed set to revolutionize the medical industry, has collapsed due to the withdrawal of its main investor, Otsuka Pharmaceuticals, which threatens the advancement of this technological development [93]. Both of these products share some commonalities: an ingestible sensor, a transmitter, and an app. In the case of Proteus, the sensor was integrated into a pill, whereas iD Capsule, as the name indicates, is a capsule made of hard gelatin. In addition, etectRx provides caregivers with a dashboard from where they can monitor individual patients as well as ingestion events across large groups of patients. Overall, in both products, as the embedded sensor moves through the patient’s digestive tract, it interacts with gastric juices and emits signals that are picked up by an external device that then transmits them to the patient’s phone. The main purpose of these digital pills is to tackle medication non-adherence, i.e., the intentional or unwitting failure to take medications as prescribed, which could be of great aid in clinical trials, for example.

In line with ingestible sensors, Atmo Biosciences’ proposal is worthy of mentioning despite not being a biosensor in the strict definition. Their product is an ingestible capsule that senses gases throughout the digestive tract of the patient for the diagnosis of gastrointestinal disorders and diseases [94]. The sensor is able to build gas profiles, including H₂, O₂, CO₂, CH₄, and temperature measurements. Through the latter, the capsule also notifies automatically once it is expelled. Data are collected by an external receptor and sent through the patient’s phone to a cloud server where it can be aggregated to build a highly valuable normative data set of gas profiles through big data and data science. Atmo claims their technique is up to 3000 times more accurate than currently used breath tests. This product has recently finished phase 1 clinical trials with positive outcomes and has a PCT pending patent [95].

However, the most representative ingestible biosensor might be one that is not yet commercialized. It was developed by a team of researchers from MIT (Massachusetts Institute of Technology) led by Dr. Timothy Lu and is called the “bacteria on a chip” because it combines living genetically modified bacteria covered by a semipermeable membrane with wireless electronics [96]. The chip has four sensing sites, or wells with immobilized *E. coli* bacteria, that emits light when it encounters blood’s heme groups in the chip. Each well is evaluated by a phototransistor that measures the amount of light produced by the bacterial cells and relays the information wirelessly to a nearby computer or smartphone. The researchers also built an Android app that can be used to analyze the data.

The proof of concept was tested in pigs, successfully detecting blood in their gastrointestinal tract [97]. Dr. Lu’s team claims the chip’s great versatility lies in the possibility of immobilizing any kind of modified bacteria, allowing the detection and sensing of a great variety of analytes and diseases. The work is undergoing a patenting process in the USA [98].

Among other kinds of biosensors currently being developed, due to their degree of innovation and transference maturity, Profusa’s Wireless Lumee^®^ Oxygen Platform [99] and Lucentix’s luciferase-based biosensors [100] stand out.

Profusa’s platform consists of an injectable oxygen micro-biosensor composed of a biocompatible hydrogel and a near-infrared oxygen-sensitive molecule with an intelligent data platform. The microsensor senses oxygen in the body based on the principle of phosphorescence quenching while a lightweight wireless adhesive patch above the skin reads the fluorescent signal from the biosensor and then transmits the data wirelessly to a tablet for real-time visualization using the Lumee app. The main intended application is the real-time monitoring of tissue oxygen in patients with potential acute and/or chronic changes in tissue oxygen levels, such as those with peripheral artery disease (PAD) and critical limb ischemia (CLI). The product achieved Conformité Européenne (CE) mark approval to start selling the platform in Europe in January 2020, while remaining limited to research applications in other markets.

Lucentix’s platform involves bioluminescent sensor proteins and low-cost electronics to achieve the measurement of precise concentrations of analytes in a single drop of blood or saliva. The bioluminescent enzyme (luciferase) is engineered to emit different colors of light in response to changes in analyte concentration. In the absence of the analyte, red light is emitted, while at a high analyte concentration, the light is blue. The system has a granted patent in the USA [101]. The system comprises a compact handheld device that carries out the readings and single-use test-strip cartridges where the drop of blood or saliva is placed. The cartridge is, in turn, placed inside the reader, and laboratory-quality results are sent in less than 5 min to the user’s phone with no sample preparation required. Lucentix was founded in 2015 at the École Polytechnique Fédérale de Lausanne (EPFL).

Another group of biosensors that piqued the public’s interest is that of tattooed biosensors. The most representative development in this group is possibly MIT’s DermalAbyss project [102], a biosensing platform that uses the skin as an interactive interface for tattoos in which traditional ink is replaced by biosensors. The proof-of-concept consisted of four biosensors: pH, UV intensity, sodium, and glucose.

## 5. Technology to the Market

Taking a promising scientific idea and converting it into a robust, reliable, and secure technology demands a huge amount of work and integrated efforts from the scientific inventors, then business and start-up founders, private capital, and regulatory parties. A lot of innovative developments in a wide range of areas, especially in health technologies, vanish every day when facing the major obstacles of final product validation such as FDA compliance, clinical trials, and user technology adoption behavior. In order to illustrate a roadmap for every technology willing to meet success, three major obstacles that any point-of-care smart device must navigate through to achieve commercialization and patient implementation are presented.

### 5.1. FDA Regulatory Compliance and CLINICAL Trials Positive Results

Prior to its insertion in the market, every product must comply with certain regulations regarding its safety and effectiveness, especially in the case of products interacting directly with the human body, such as biomedical biosensors. Each country has its own institutions in charge of regulations and supervision of the commercialization of these products, ultimately looking out for the consumers’ safety.

A thorough analysis of the regulations merits a complete publication by themselves due to their vastness and intricacies. Instead, through a brief study of the regulatory compliance certification process, the main obstacles to the transfer to the market are elucidated. Considering that nearly 70% of medical technology companies with more than $1 billion in annual revenue are based in the United States, the focus will be on this market. However, the reader can refer to Gupta’s work (Medical Device Regulations: A Current Perspective) for a broader panorama [103], or to Manita’s work (Regulation and Clinical Investigation of Medical Devices in the European Union) for an insight into the EU’s regulations [104].

In the United States, medical devices are regulated under the Federal Food, Drug, and Cosmetic Act by enforcement of the Food and Drug Administration (FDA). Within the FDA, the Center for Devices and Radiological Health (CDRH) is the institution responsible for pre- and post-market supervision of medical devices.

Currently, there are two main pathways manufacturers can follow to obtain market approval or clearance for their products. One path involves carrying out extensive clinical trials and submitting a pre-market approval (PMA), whereas the other path requires the submission of a 510(k) notification. The former is substantially costlier and takes more time compared to the latter. A third pathway is available for devices aiming to treat or diagnose conditions affecting 4000 or fewer individuals under a “Humanitarian Device Exemption” (HDE).

A 510(k) requires the submitter to demonstrate that the new device is “substantially equivalent” to a legally marketed device. Thus, substantial equivalence enables a manufacturer to market a new device without presenting safety or effectiveness data, though they are still required to comply with regulations on manufacturing, labeling, surveillance, device tracking, and adverse event reporting. A fee must be paid for each submission with differentiation depending on the size of the company requesting the review.

In recent years, the FDA has made numerous changes to its review system, attempting to reach “the least burdensome approach in all areas of medical device regulation”, i.e., “*the minimum amount of information necessary to adequately address a relevant regulatory question or issue through the most efficient manner at the right time*” [105]. For example, the Safety and Innovation Act allowed collaboration with foreign government regulations, the classification of low-to-moderate risk devices as Class I or II while bypassing the 510(k) and sped up review times while the 21st Century Cures Act expanded the FDA’s least burdensome approach, further facilitating for devices to obtain 510(k) exemptions. Additionally, the FDA provides clear advice on its web page on how to properly market a device, under a “Comprehensive Regulatory Assistance” section [106], in order to further help manufacturers.

These modifications resulted in significant growth in the amount of marketed MDs, though not without controversy as there are critical reports, medical journal articles, and even testimonies before Congress stating that the FDA’s current approach causes a great oversight, ultimately endangering users. Still, the FDA’s requirement for reasonable assurance of safety and effectiveness as opposed to the safety and performance standard required in most other countries results in an overall need for more clinical data and larger clinical studies to support U.S. marketing approval [107].

Zuckerman et al. evaluated recalled high-risk devices from 2005 to 2009 and found that around 78% of them were cleared through the faster 510(k) process or exempt from regulatory review [108]. While this does not increase the time-to-market, it does affect the success of new devices as a wrongly cleared device that ends causing harm to users or that fails to deliver the intended treatment or diagnosis, being recalled, could affect the user’s perception of all devices of the same kind, for example, biosensors.

While a lax regulatory system can ease the transference of technology, companies should not exploit loopholes or fast pathways without significant proof of safety and effectiveness in a race to reach the market as this could lead to an overall negative impact with the potential to set back the whole industry due to poor adoption of the products of the same class that reaches the market.

In analyzing the time-to-market of the companies and products mentioned in this review, apparently, the main bottleneck in the transference process is in the clinical trials stage. Currently, many manufacturers receive approval based on early data and commit to performing post-approval trials. This approach finds its reasons in the fact that many device trials assess iterative improvements and that device designs usually change during or in between trials. However, these commitments and post- marketing requirements often remain incomplete for years after approval [109]. For example, in their review, Rathi et al. found that among high-risk devices that received pre-marketing approval between 2010 and 2011, only 13% of initiated post-marketing studies were completed between 3 and 5 years after FDA approval [110].

Clinical trials are expensive, complex, and have to be carefully designed in order to ensure the validity of the data they generate. The specific details of the study's design will largely impact the time and feasibility of their completion and the cost of a medical device becoming cleared to market. Medical devices trialshave added difficulties [111]. Specific barriers and challenges include the difficulty of conducting blind trials and choosing appropriate comparison groups. Another challenge that medical device trials face is that of the learning curve that some of these devices may have, and that may even be steeper in some cases, as this is something that both patients and clinicians participating in the trial must overcome.

It became clear that faster pathways for safe and useful trials must be achieved. While regulations and institutions that enforce them have come a long way in trying to improve the overall process, there is undoubtedly a lot to be developed further. A balance must be achieved, where companies can be enticed to develop new technologies with the possibility to market them in the fastest and cheapest way possible while ensuring the consumers, safety and effectiveness.

### 5.2. Technical Limitations of the Technology

It is of utmost importance to consider and analyze point-of-care smartphone-based biosensors free of the hype that oftentimes surround them and to take into account their technical limitations. For example, while ubiquitous, smartphones’ cameras are not specifically designed for close-up determinations and may require some sort of adapting hardware to ensure the image is taken with the minimum necessary quality. The great variety in optical sensors, lenses, and image processing software between phone manufacturers makes the standardization of the results slightly trickier and this results, in turn, in most of these diagnoses being merely qualitative. One example of this limitation happened to Priye et al. [51] when they proposed their device, where complex auxiliary electronics were necessary for its operation. All these complicated setup and add-ons cast doubt around how purely smartphone-based a device can be since there are many unavoidable requirements that biological and chemical reactions demand in order to provide us with high-quality results, requirements that, at the same time, force us to implement more sophisticated hardware setups that leaves smartphones as merely a detector or just another piece of the system.

Another limitation resides in the electrical power of smartphones. Battery autonomy is always a pain point for users, having to charge their devices practically every day. While significant improvements in both batteries and processors were made, it is still a limiting factor to consider when thinking of multiple determinations in the POC. Moreover, the limited power of a smartphone’s battery sets a limit in the complementary hardware that is sometimes needed for some smartphone-based biosensors to operate, for example, in the case of some electrochemical devices. This situation can be seen in electrochemical smart-biosensors, where specific detection and analyzing requirements are needed for high-quality results that sometimes exceed smartphone capabilities. A clear example is the one mentioned in this review proposed by Shin Low et al. [33], in which they coupled the smartphone to a circuit board composed of several main components, including a Bluetooth module, microcontroller unit, digital/analog converter, potentiostat module, and power management module. Main measurements and detection were performed because of this circuit board leaving the analyzing part to the smartphone, something that can be conducted with a personal computer or another analyzer. It is worth mentioning that the OTG USB port was used as a power supply, which limits the number of determinations to the phone-battery capacity. Hence, it can be concluded that outstanding advantages of smartphone use in biosensor devices are sometimes shady and suggests that specific and dedicated hardware can make a true difference in order to achieve efficient and robust POC results. Added to this, the standard for different interfaces in smartphones, for example, the 3.5 mm audio jack and USB connector as well as Bluetooth antennae, have limits in the amount of power each interface can handle.

### 5.3. User Adoption Limitations

Finding out and recognizing the needs and acceptance of final users is the beginning of any business based on technology development, and this understanding is crucial for finding a path for future advances. Thus academicians must be interested in the factors that drive users’ acceptance or rejection of technologies. Although there are several social and psychological theories that attempt to explain the motivators and inhibitors that drive user acceptance of certain technology [112], modern technology and product development are based on the need for commercial profits by satisfying user needs. Technologists, designers, and psychologists moved by the spirit of reaching a certain balance between commercial and user-caring strategies developed a working methodology called “User-Centered Design” (UCD). Proposed by Donald Norman and Stephen Draper in 1986 [113], UCD is an iterative design process in which designers focus on the users and their needs in each phase of the design process. At UCD, the design teams involve users in the design process so that the products created are truly usable and accessible to them. Therefore, the development team should include professionals from across multiple disciplines (e.g., ethnographers, psychologists, software and hardware engineers), as well as domain experts, stakeholders, and the users themselves. Experts may carry out evaluations of the produced developments, using different guidelines and criteria.

Pure academic researchers and certain technologists sometimes conduct their research guided only by scientific and theoretical motivations. They based their development on doing the best science they can, but this is not always enough. For a consistent market landing and establishment, UCD strategies should be applied in technology development by academia. When the research team brings the users into every stage of the development and research process, effort and other resources are invested into a powerful way of finding out what works well, what does not, and why. Users are an early-warning system that can be used to course-correct and fine-tune proposed devices. UCD exposes many aspects—positive and negative—that the research team may have overlooked regarding such vital areas as usability and accessibility. That is why it is so important to understand how powerful the benefits of a user-centered design approach are. In this review, many of the proposed devices did not use real samples for performance evaluation (see Table 1, Table 2 and Table 3), or sometimes target analyte lacked true clinical relevance. Lastly, it can be seen that most of the reviewed researches lacked cost-efficiency analysis and evaluation of the real need for a smartphone-based device, which in many of the cases did not add extra advantages in portability, ease of use, and cheaper setups. Many times, fully developed devices are then confronted with the reality of the users, and many times, they lead the development to a failed landing in the market.

Another inhibitor of technology adoption in the information and internet era is user concern by privacy and security [114]. Two main issues regarding security and privacy in smart devices are the complex interactions that take place during the typical use of smart healthcare solutions (e.g., patients/users with their caregivers/medical professionals), along with the sensitive nature of the handled data. These factors need the integration of strong and reliable security mechanisms and privacy provisions, including clear authentication and authorization services, for the protection of user sensible data. Smart devices are designed mainly considering low-cost, low-energy usage, ease of setup and use, and interconnection, but not security. Since health monitoring systems may include sensitive data, it is important to protect them from possible attackers. All adopted security and privacy mechanisms must be refined to accomplish the necessary requirements. A key feature of these mechanisms will be their capability to adapt in real-time to several conditions of usage and requirements (e.g., context, privacy preferences, risk profile, and others). Those that can fulfill this fast adaptability and strong security features will be more prepared for faster user adoption and its way to market establishment.

Therefore, after all these critical limitations are seen, this scenario leaves a question of whether there is a paramount innovation that relies on smartphone-based devices or it is only a popular research trend that needs to be properly revised in order to produce more “ready to market user-centered devices”.

## 6. Conclusions

A detailed review of the different types of smartphone-based POC devices was presented, and the ones that reached the commercial stage were particularly analyzed. The different regulations that the devices must comply with were shown, and some observations, which reinforce or limit the passage of the developments reported in the bibliography to the commercial stage were also presented. The vast literature reviewed demonstrates a large number of such devices, the acuity of some, and how useful they can be to patients or physicians on the “battlefield”.

In an analysis of the publications that show that they can advance towards a commercial-stage through patents or related patents, it was found that in the analyzed period, there are different percentages of patenting according to the technologies, with optics being the most patented proportionally, followed by paper-based devices. This may be due to the rapid evolution of smartphones both in processing power and accessories such as cameras and light sensors.

It was interesting to discover that by extending the study period to 10 years, a marked increase in application patents is clearly noticeable towards the years 2014 to 2016 and then a marked decrease in the last 6 years. The reasons can be many, but perhaps the most important would be the difficulty of technology transfer and adoption by end-users. Reviewing the different technologies available already in the commercial stage, it was observed that they are still scarce despite the great development these technologies have experienced due to the capabilities of smartphones. As mentioned, there is a delay between the report of the technology through a publication and its appearance on the market; therefore, this allows us to foresee a large increase in the coming years. However, it is clear that more work needs to be conducted to strengthen the transfer of smartphone-based biosensor technology to help in the daily fight for people’s health.

## Figures and Tables

**Table 1:** Smartphone-based electrochemical biosensors compared with benchtop techniques.

Application	Biosensor Type	Evaluated in Real Samples?	Pat. Nº, Year, State	Improvements of Smart Sensor vs. Benchtop Techniques	Refs.
Secretory leukocyte protease inhibitor (SLPI) but can be applied to different applications	Immunological	No. Tested in solutions of different concentrations of the biomarker secretory leukocyte protease inhibitor (SLPI)	US11166653B2, 2016/2021 [24]	Electronic module containing a low-power potentiostat that interfaces efficiently with a wide variety of phones through the audio jack to obtain power and communicate. The system uses a microcontroller. Total power consumption: 6.9 mW. Compared with a commercial potentiostat: current from ±300 pA to ±20 µA with a 100 kΩ gain. It can be used to obtain voltammograms. The platform can be used with different brands of smartphones and allows the use of electrochemical biosensors for different applications.	[25,26]
			US20210087614A1, 2019 Pending [27]		
Blood β-ketone (blood β-hydroxybutyrate)	Enzymatic: β-hydroxybutyrate dehydrogenase method	Yes. Tested in finger blood		Electrochemical dongle, which is powered by the smartphone through an OTG. It takes chronoamperometric measurements of blood ketone. Linear regression coefficient of 0.987 for a range of 0 to 4 mmol/L of blood β-hydroxybutyrate. The authors were able to demonstrate that the preciseness and stability of the measured data are highly reliable and applicable for clinical use.	[28]
For protein detection: bull serum albumin (BSA) and thrombin	Immunological for BSA detection and Enzymatic for Thrombin detection	No. Tested with solutions of different concentrations of BSA and thrombin		Portable transducer and a handheld detector connected via Bluetooth to the smartphone. The detector can perform electrochemical impedance spectroscopy (EIS) (10 Hz to 10 kHz). The system can detect very low concentrations of BSA (1.78 µg/mL) and thrombin (2.97 ng/mL). They related the charge transfer resistance (Rct) with the concentration of BSA or thrombin. The smartphone delivers control commands, receive data signals, and display the Nyquist graph. A designed Android App serves as an interactive interface between the users and the biosensor system. It allows the use of other electrochemical biosensors.	[29]
Glucose concentration	Enzyme-carbon composite pellets	No. Tested with solutions of different glucose concentrations	US20210270766A1, 2018 Pending [30]	Electrochemical sensor strips consist of carbon electrodes and a second part is composed of the carbon paste GOx biosensor, which can be replaced in each measurement. The biosensor is a compact carbon/GOx/rhodium pill. Measurement compartment: 3D-fabricated smartphone case with a permanently-attached passive sensor strip and a compartment where the biosensor magnetic pellet is placed for each measurement. They developed a portable potentiostat (Texas Instruments CC2541 BLE System on-Chip) communicated wirelessly with the smartphone. Android-based smartphone application developed.	[31]
Alcohol in whole blood samples	Enzymatic: two enzymes are used, HRP and alcohol oxidase	Yes. Tested with whole blood		The system combines a three-electrode microfluidic chip with a secondary compact PCB module as a µPotentiostat. Chronoamperometric and CV measurements. Communicated with the smartphone via USB. The novelty of the system is the reusable biosensor concept. Two enzymes, HRP and alcohol oxidase, are immobilized via in situ electrodeposition of a calcium alginate hydrogel for selective ethanol detection. A constant potential of 0 V was applied between WE and Pt RE. The smartphone acts as a simple graphical interface and for cloud connectivity.	[32]
Cancer biomarker microRNAs (miRNAs)	Genetic: Tris(2-carboxyethyl)phosphine hydrochloride (TCEP)-treated ssDNA probe drop casted onto an rGO/Au composite-modified WE	No. Tested with miR-21 spiked artificial saliva		The system presents a circuit board as the potentiostat, powered through smartphone On-The-Go (OTG) port and a graphene oxide/gold composite-modified electrode as the biosensor. The circuit board communicates via Bluetooth with the smartphone. A specially designed Android application shows the results. The detection is facilitated via a synthetic ssDNA probe immobilized onto the GO/Au electrode. Good linearity (R^2^ = 0.99) for the detection of 1×10^−4^ M to 1×10^−12^ M of [miR-21]. The sample must be incubated at 40 °C for 1 h for hybridization before electrochemical measurement.	[33]
Reactive oxygen species (ROS) for COVID-19 detection	MWCNTs on the tip of steel needles of 3 electrodes	Yes. Tested in Fresh sputum or bronchoalveolar lavage fluids	US11181499B2, 2017/2021 [34]	The system includes a previously patented electrochemical ROS/H2O2 system consisting of an electrochemical readout board (+/−0.8 mV, 100 mV s^−1^, and a sensing disposable sensor. The group presented an application Patent in 2020 for the electrochemical approach to detect COVID-19, which was granted in 2021.	[35]
			US11047824B2, 2020/2021 [36]		
RNA from SARS-CoV-2 virus	Genetic: The sequences were provided by the Chinese Center for Disease Control and Prevention (CDC)	Yes. Tested with extracts from SARS-CoV-2-confirmed patients and recovered patients		It is an ultrasensitive electrochemical biosensor for the detection of the RNA of SARS-CoV-2 by using a smartphone. They used a super sandwich-type recognition strategy without the need for nucleic acid amplification and reverse transcription. For this biosensor, only two copies (10 μL) of SARS-CoV-2 were required per assay to detect a positive sample. Calibrated with concentrations between 10^−17^–10^−12^ M, LOD: 3 aM. LOD of the clinical specimen: 200 copies/mL, which was the lowest LOD among the published RNA measurement of SARS-CoV-2 at this moment	[37]

**Table 2:** Smartphone-based optical biosensors compared with benchtop techniques.

Application	Biosensor Type	Evaluated in Real Samples?	Pat. Nº, Year, State	Improvements of Smart Sensor vs. Benchtop Techniques	Ref.
H_2_O_2_ , Glucose and Catechol biosensor	Enzymatic: GOX and tyrosinase over poly(aniline-co-anthranilic acid)	Yes. Food and pharmaceutical samples		Polymeric substrate material and image processing software provided a great correlation with benchtop techniques and higher LOD.	[44]
HIV and Hepatitis B biosensor	DNA/RNA-linked biosensor	Yes. Plasma samples	WO2014089700A1, 2013 Pending [45]	They were able to detect between 10^3^ to 10^9^ copies/mL over a 20 µL sample and differentiate patients with HIV from those with HBV on the mono-infection assay and multiplexed detection of both of them in a co-infection assay. The results were quite well-correlated compared to benchtop equipment measurements.	[46]
Hemoglobin and HIV biosensor	Immunosensor	Yes. Blood samples	WO2016025698A1, 2014 Pending [47]	It consists of a combined pure optical assay and an immunoassay at the same time, and in the same device, without a difficult procedure for handling samples and reagents. The results are in good agreement with their commercial equivalents supported by smartphone technologies.	[48]
*E. coli* and *S. typhimurium* biosensor	Immunosensor	No		For the first time, a device capable of detecting two genetically related bacteria within a single sample drop is reported, with a LOD of 10^−2^ CFU/mL, in a fairly short time (12 min), and with a good consistency in comparison with the results obtained in laboratory experiments.	[49]
Zika, Dengue, Chikungunya detector	DNA/RNA-linked biosensor	No. Tested in artificial blood, urine, and saliva samples	US20160025630A1, 2014 Pending [50]	Detection technique that involves quenching of unincorporated amplification signal reporters (QUASR). Distinctively to other reported LAMP detection modalities, QUASR offers very bright signals, reduces the detection of false-positive amplification, and offers the ability to multiplex two or more targets per reaction. These features can highly reduce reagent costs and dilution needs when sample volume is limiting. A personalized smartphone application (app) controls the isothermal heating module and a LED excitation module via Bluetooth. The app processes images through a novel detection algorithm for multiplexed QUASR assay signals with greater accuracy than conventional image analysis software.	[51]
HIV1-p17, hemagglutinin (HA), and dengue virus type I detector	Bioluminiscent reporter	Yes. Blood plasma samples	WO2019038375A1, 2018 Pending [52]	The design shows to be an attractive analytical platform for point-of-care antibody detection that dispenses with liquid handling steps that are related to the major issues in immunoassays.	[53]
Inflammation and cell viability biosensors	Bioluminiscent reporters	No. Simulated proinflammatory and toxic samples.	US20120045835A1, 2009 Pending [54]	A limit of detection for tumor necrosis factor (TNFα) of 0.15 ± 0.05 ng/mL was achieved. This proposal promises to be a useful platform to preliminary screen environmental samples or other types of compounds for on-site detection.	[55]
Hemoglobin sensor	Label-free detection	No. Simulated samples.	US8861086B2, 2014 [56]	It stands out for its compact size, portability, low cost, the efficiency of optical spectroscopy for quantitative measurement, and ease of data collection, management, and computation.	[57]
			US20160296118A1, 2015 Pending [58]		
Bovine immunoglobulinG (IgG)	Immunosensor	No. Spiked buffer solution of IgG protein	US20190025330A1, 2917 Pending [59]	In addition to the ability to detect immunoglobulins G, the device can be applied to the sensing of other analytes by properly functionalizing the gold film. The results and sensitivity obtained were comparable to commercial SPR instruments, so being a portable SPR system, it makes it an extremely useful device.	[60]
Chloride, sodium, and zinc in sweat	Fluorescence	Yes. Sweat	US20210145352A1, 2018 Pending [61]	Through an ultrathin, skin-compatible adhesive layer, the device allows sweat to be collected and distributed to different areas with fluorescent reagents. The device makes it possible to quantitatively determine, in a simple and low-cost device, several biomarkers of sweat at the same time.	[62]
Prostatespecific antigen (PSA)	Fluorescence	No. Spiked solution with PSA	US20120141746A1, 2009 Pending [63]	The device allows, through simple steps, to quantify different concentrations of PSA by means of fluorescence measurement with a smartphone. This sends the data to the cloud for processing and gives a result in about 1 min. It is not a practical device since it needs an objective lens (magnification 40×) to be able to capture the images with the smartphone.	[64]
			JP2008128677A, 2006 Pending [65]		
			WO2017141503A1, 2016 Pending [66]		

**Table 3:** Smart paper-based biosensor devices classified according to the principle and the type of detection.

Applications	Biosensor Type	Evaluated in Real Samples?	Pat. Nº, Year, State	Improvements of Smart Sensor vs. Benchtop Techniques	Ref.
µCTX-II in urine	Immunological	No. Tested with artificial urine solution (AUS) with the same composition as real urine	US20180371529A1, 2015 Pending [72]	Effective smart optical biosensor, highly correlated with benchtop techniques and higher LOD for the use in patients with complications of renal insufficiency and also for the diagnosis and/or prognosis of osteoarthritis.	[73]
Hemoglobin	Colorimetric	Yes. Finger-pricked blood	WO2021019553A1, 2019 Pending [74] WO2021019552A1, 2020 Pending [75]	Fast, sensitive, and specific device for the detection of anemia with good correlation with the results of an automated hematology analyzer and on par with other POC test platforms. The results differ from the pathological estimates within the range of 0.5 g/dL for all severely anemic samples and <1.5 g/dL for the rest of the samples.	[76]
Urinary microbial ATP	Bioluminescent	No. A urine sample inoculated with *E.Coli* was used to simulate a urinary tract infection.	US8642272B2, 2014 [77]	First device bioluminescent on paper for the detection of low-cost ATP, based on the reaction of Luciferase/D-Luciferina that exploits the smartphone camera as a detector. The ATP sensing paper includes an Innovator Lyophilized “Nano-Lantern” With Reaction Components. The mentioned patent does not correspond to the device but is related to its manufacturing materials.	[78]
Human IgM and IgG	Immunological	Yes. Human serum	US20210382048A1, 2021 Pending [79]	This paper device has a detection limit of 100 fg/mL demonstrated for the biomarkers of the IgG and IgM protein, which is higher than the one achieved with a traditional Benchtop ELISA test. It is also a much faster method (<5 min), portable, resistant, stable, and low cost, which uses serum without sample preparation and can be easily discarded.	[80]
SARS-CoV-2	Genetic: AuNPs capped with highly specific antisense oligonucleotides (ssDNA)	Yes. Samples collected from Vero cells infected with SARS-CoV-2 virus and clinical samples	US20210388454A1, 2020 Pending [81]	This device can successfully and precisely distinguish the positive samples from Covid-19 from negatives, with sensitivity and specificity of almost 100%. It also presents sensing feasibility even for virus genomic mutation events due to the use of AuNPs, covered with highly specific antisense oligonucleotides (SSDNA) that are simultaneously directed to two separate regions of the same SAR-CoV-2 N gene	[82]
Cotinine in Urine	Immunological-Electrochemical	Yes. Urine samples of smoker and non-smokers patients	WO2019139537A1, 2019 Pending [83]	A simple lateral flow competitive immunochromatography was successfully integrated with the AgNP/HRP/AuNP-modified electrode. Immunoreaction can be monitored by either electrochemical measurement or wireless detection. Wireless sensing was realized for cotinine in the range of 100–1000 ng/mL (R^2^ = 0.96) in PBS medium. For 1:8 diluted urine samples, the device differentiated positive and negative samples and exhibited cotinine discrimination at levels higher than 12 ng/mL.	[84]
IL-6 levels in blood and respiratory samples	Immunological	Yes. Human blood and bronchial aspirate samples	WO2021048087A1, 2019 Pending [85]	Paper immunosensor interfaced with a smartphone that generates intense colorimetric signals when the sample contains ultralow concentrations of IL-6. The device combines a paper-based signal amplification mechanism with polymer-filled reservoirs for dispensing antibody-decorated nanoparticles and a bespoken app for color quantification. Semi-quantitative measurements of IL-6 can be facilitated in 10 min with a LOD of 1.3 pg mL^−1^ and a dynamic range of up to 102 pg mL^−1^ in diluted blood samples.	[86]
